# Neuroinflammation is independently associated with brain network dysfunction in Alzheimer’s disease

**DOI:** 10.1038/s41380-022-01878-z

**Published:** 2022-12-06

**Authors:** Fangda Leng, Rainer Hinz, Steve Gentleman, Adam Hampshire, Melanie Dani, David J. Brooks, Paul Edison

**Affiliations:** 1 Department of Brain Sciences, Imperial College London, London, UK; 2 Department of Neurology, Peking University First Hospital, Beijing, PR China; 3 Wolfson Molecular Imaging Centre, University of Manchester, Manchester, UK; 4 Institute of Clinical Medicine, Aarhus University, Aarhus, Denmark; 5 Institute of Translational and Clinical Research, University of Newcastle upon Tyne, Newcastle, UK; 6 School of Medicine, Cardiff University, Wales, UK

**Keywords:** Neuroscience, Diseases

## Abstract

Brain network dysfunction is increasingly recognised in Alzheimer’s disease (AD). However, the causes of brain connectivity disruption are still poorly understood. Recently, neuroinflammation has been identified as an important factor in AD pathogenesis. Microglia participate in the construction and maintenance of healthy neuronal networks, but pro-inflammatory microglia can also damage these circuits. We hypothesised that microglial activation is independently associated with brain connectivity disruption in AD. We performed a cross-sectional multimodal imaging study and interrogated the relationship between imaging biomarkers of neuroinflammation, Aβ deposition, brain connectivity and cognition. 42 participants (12 Aβ-positive MCI, 14 Aβ-positive AD and 16 Aβ-negative healthy controls) were recruited. Participants had ^11^C-PBR28 and ^18^F-flutemetamol PET to quantify Aβ deposition and microglial activation, T1-weighted, diffusion tensor and resting-state functional MRI to assess structural network and functional network. ^11^C-PBR28 uptake, structural network integrity and functional network orgnisation were compared across diagnostic groups and the relationship between neuroinflammation and brain network was tested in 26 Aβ-positive patients. Increased ^11^C-PBR28 uptake, decreased FA, network small-worldness and local efficiency were observed in AD patients. Cortical ^11^C-PBR28 uptake correlated negatively with structural integrity (standardised *β* = −0.375, *p* = 0.037) and network local efficiency (standardised *β* = −0.468, *p* < 0.001), independent of cortical thickness and Aβ deposition, while Aβ was not. Network structural integrity, small-worldness and local efficiency, and cortical thickness were positively associated with cognition. Our findings suggest cortical neuroinflammation coincide with structural and functional network disruption independent of Aβ and cortical atrophy. These findings link the brain connectivity change and pathological process in Alzheimer’s disease, and suggest a pathway from neuroinflammation to systemic brain dysfunction.

## Introduction

The pathological hallmarks of Alzheimer’s disease are extracellular β-amyloid (Aβ) plaques and intraneuronal neurofibrillary tangles (NFT) consisting of tau protein. The ‘amyloid cascade hypothesis’ posits initial Aβ aggregation leads to intracellular tau phosphorylation, tangle formation and neuronal dysfunction [1]. Both Aβ and NFT are associated with neuroinflammation in the form of activated microglia, which might mediate their neurotoxicity [2]. Genome-wide association studies (GWAS) have found that innate immune-related genes such as TREM2 (Triggering Receptor Expressed on Myeloid Cells 2) are associated with the risk of sporadic AD, independent of apolipoprotein E4 status [3]. It is now well-accepted that neuroinflammation plays a significant role in the process of neurodegeneration [4].

Brain network disruption is considered responsible for cognitive impairment in AD [5]. Diffusion tensor imaging (DTI) and resting-state functional MRI (rs-fMRI) have enabled evaluation of brain network structural integrity and functional organisation in vivo [6, 7]. Recent studies have revealed disruption of structural and functional connectivity early in the Alzheimer’s trajectory and they are associated with cognitive impairment [8, 9].

However, the pathological events disrupting structural and functional brain networks in the Alzheimer’s spectrum remain to be clarified. While there is imaging and pathological evidence that tau deposition is associated with white-matter degeneration [10, 11], the influence of amyloid deposition on brain connectivity in Alzheimer’s disease remains controversial [12, 13]. More importantly, the influence of neuroinflammation on brain connectivity needs further evaluation, given its close relationship with Alzheimer’s pathologies and the supportive function of homoeostatic microglia for neurones in healthy human brain [14]. We hypothesised that levels of microglial activation are responsible for network structural integrity damage and functional organisation disruption in AD. To test the hypothesis, we analysed the association between ^11^C-PBR28 uptake and the brain’s structural and functional network measures in Aβ-positive patients while controlling for amyloid deposition and grey-matter integrity.

## Patients and methods

### Study cohort recruitment and patient consent

The cross-sectional study was approved by the London Riverside Research Ethics Committee, National Health Research Services, Health Research Authority, UK. Approval for administration of PET tracers was obtained from the Administration of Radioactive Substances Advisory Committee (ARSAC). All participants in the study gave written informed consent.

The inclusion criteria of the study were: (1) diagnosis of MCI or AD according to NIA-AA (National Institute of Aging and Alzheimer’s Association) criteria or normal cognitive function for healthy volunteers (HCs) [15, 16]; (2) 50–85-year old; (3) Mini-Mental State Examination (MMSE) score 26-30 for HCs, >24 for MCI and >15 for AD patients. To further ensure the participants are representative of patients within Alzheimer’s continuum, only patients (MCI and AD) who were positive on ^18^F-Flutemetamol PET scans were included in the analysis. Healthy volunteers who were amyloid-PET negative were included as reference to assess neuroinflammation and network changes in the patients. 12 of the 25 MCI and 14 of 16 clinically diagnosed AD patients were considered Aβ-positive (Aβ+) based on increased ^18^F-flutemetamol SUVR (>1.46) in the composite region (frontal, parietal, lateral temporal, anterior and posterior cingulate cortices) defined by Thurfjell et al. [17].

Candidates with the following conditions were excluded: (1) major depression, schizophrenia, or schizoaffective disorders; (2) history or signs of other neurological diseases; (3) malignancy within the last 5 years; (4) contraindications for MRI scanning; (5) homozygous for the 147 Thr/Thr single nucleotide polymorphism of the 18 kDa translocator protein (TSPO) gene (low affinity binders for 11C-PBR28).

### Image acquisition

#### MRI

MRI were acquired with a 3 Tesla Siemens Verio scanner using a 32-channel head coil. T1-weighted images were acquired with TR = 2300 ms, TE = 2.98 ms, FA = 9°, TI = 900 ms, 1 × 1 × 1 mm voxel. Diffusion tensor images were acquired in 64 diffusion directions using an EPI sequence: TR = 9000 ms, TE = 99 ms, 2 × 2 × 2 mm voxel, b-value = 1000 s/mm^2^. Resting-state functional scans (rs-fMRI) were acquired by an EPI sequence over 10 min with the following parameters: TR = 2000 ms, TE = 30 ms, FA = 80°, 3 × 3 × 3 mm voxes. A total of 300 volumes were acquired per scan.

#### ^11^C-PBR28 PET

^11^C-PBR28 was manufactured at the Imanova Centre for Imaging Sciences in London, and a Siemens Truepoint PET/CT scanner was used for imaging. 330.9(±30)MBq ^11^C-PBR28 in 20 ml normal saline was injected intravenously. 90-min list mode 3D-dynamic acquisition data were rebinned using the following time frames: 8 × 15 s, 3 × 60 s, 5 × 120 s, 5 × 300 s and 5 × 600 s. Filtered back projection with attenuation correction and 5 mm Gaussian filter was used in reconstruction.

#### ^18^F-flutemetamol PET

^18^F-flutemetamol was manufactured by GE Healthcare, Amersham, UK. Scans were performed at the Imperial College Clinical Imaging Facility with a Siemens Biograph 6 scanner. 183.4(±5.3)MBq ^18^F-flutemetamol was injected intravenously in 8 ml saline followed by 10 ml saline flush. Data were acquired in 3D-list mode from 90 to 120 min following injection (6 × 5 min frames). Image reconstruction was performed by filtered back projection with attenuation correction and post-reconstruction 5 mm Gaussian smoothing.

### Image processing

#### PET

PET image processing was performed using Analyze AVW 11.0 and Statistical Parametric Mapping 8 (SPM8, Wellcome Trust Centre for Neuroimaging, UCL). PET images were co-registered to T1 MRI and transformed into Montreal Neurological Institute (MNI) space for voxel-wise analysis. Individualised atlases in PET space were generated based on Hammer’s probabilistic atlas. 60–90 min standard uptake value ratio (SUVR) of ^11^C-PBR28 and 90–120 min SUVR of ^18^F-flutemetamol were calculated using cerebellum grey-matter as the reference region, as has been validated by previous studies [17, 18].

#### MRI

Cortical thickness was measured on T1-weighted images using FreeSurfer (V6, Harvard Medical School; surfer.nmr.mgh.harvard.edu) [19]. Diffusion tensor images were denoised, motion, distortion and eddy-current corrected, and brain-extracted using FSL software (FMRIB Software Library, v6.0) [20]. Diffusion tensors were then fitted into respective maps. Fractional anisotropy (FA) maps were computed using the FSL-TBSS pipeline. A threshold of 0.2 was used to create FA skeletons. The ICBM-DTI-based white-matter atlas was used to sample FA values from tracts of interest (TOIs).

rs-fMRI was pre-processed by FEAT tool in FSL. Briefly, the first five volumes of rs-fMRI images were discarded to allow magnetic field stabilisation. The remaining volumes were realigned, slice-time corrected and smoothed (4 mm Gaussian kernel). Functional images were registered to T1-weighted images and transformed to MNI space. A combined nuisance regression and independent component analysis (ICA) denoising was performed, which included high-pass temporal filtering (>0.01 Hz), followed by removal of independent components identified as noise, and variance explained by motion parameters and their first-order temporal derivatives.

Construction of functional connectivity matrices was performed by GRETNA [21] using the anatomical automatic labelling atlas (AAL-90). A 90 × 90 z-transformed connectivity matrix was calculated for each participant. Binarised connectivity matrices were created with a network sparsity threshold of 0.2, based on the following reasons: (1) the network metrics trended to stabilise after sparsity level of 0.2 was reached, (2) the metrics computed under 0.2 threshold better distinguished diagnostic groups (AD and HCs). To avoid bias associated with a specific sparsity threshold, the area under curve (AUC) of each network metric under sparsity threshold from 0.05 to 0.50 were calculated (Supplementary Fig. 1). To investigate the global functional network organisation, global efficiency (network integration), local efficiency (network segregation), and small-worldness were computed (The biological and technical details of the network metrics are explained in Supplementary Materials).

### Statistical analysis

Statistical analyses of numeric variables were performed with R (R Core Team (2019), Vienna, Austria). Cross-group difference was tested using one-way ANOVA and Kruskal-Wallis tests, and post hoc 2-sample t-tests or Mann-Whitney tests were performed where appropriate. Equal variance was tested and statistical inferences were made with or without equal variance assumption accordingly. Voxel-wise comparisons of PET images were performed using SPM8. Clusters >50 voxels (*p* < 0.05) surviving a false discovery rate (FDR) correction (*p* < 0.05) were considered as significant. Voxel-level analyses of FA skeletons were performed with the FSL randomise tool, and the threshold-free cluster enhancement (TFCE) algorithm was used for multiple comparison corrections [22].

Further, Aβ-positive patients (AD and MCI, *n* = 26, MMSE = 25.3 ± 3.75) were grouped together as these patients were considered to represent the Alzheimer’s spectrum [23], and were used in linear regression analyses.

To represent global structural network integrity, a principal component analysis (PCA) was first performed on FA values of all tracts of interest (TOIs), and the first two principal components (PCs, explaining 40% and 9.7% of total variances respectively) were further analysed. The first PC of ^11^C-PBR28 and ^18^F-flutemetamol SUVR (explaining 78% and 96% of variance respectlively) in the bilateral frontal, temporal, parietal, occipital, anterior and posterior cingulate cortices were used to represent global neuroinflammation and Aβ deposition, respectively, while average cortical thickness was used to reflect cortical atrophy. The other PCs explaining less than 5% total variance were not included.

Regression analyses were performed to interrogate the relationship between PET measures, network metrics and cognition. Age and gender were adjusted in the above regression analyses. Robust regression method was used when assumptions of normal residual distributions couldn’t be made, and permutation tests were performed to confirm the significance of estimated coefficients.

To examine the association of tracer uptake in different ROIs with network structural integrity and to identify most relevant regions, sparse canonical correlation analyses (SCCA) were performed between FA values and ^11^C-PBR28/^18^F-flutematemol uptake, using the penalised multivariate analysis (PMA) package in R [24]. The technical details are briefly described in Supplementary Materials. A range of λ (lasso penalty, 0.10–0.70 incrementing at 2/3) was tested and the model fits were inspected. For simplicity, the statistical test results and canonical weights were presented with the λ giving best fit.

To further elucidate the direct influence and indirect effect of neuroinflammation via structural network integrity on functional network organisation, linear mediation analysis was performed with 5000 Monte Carlo simulations of quasi-Bayesian approximations [25].

## Results

### Demographics

14 AD, 12 MCI and 16 HCs were included in the current study, with their demographics summarised in Table 1. The mean ages (±SD) of the AD and MCI patients were 73.4 ± 7.7 and 75.8 ± 7.6, which were older than HCs (63.1 ± 8.2). The mean MMSE were 23.0 ± 3.6, 28.0 ± 1.5 and 28.7 ± 1.7 for the AD, MCI and HCs, respectively.

**Table 1:** Demographics of the study cohort.

	AD	MCI	HC	ANOVA
Cases	14	12	16	–
Age	73.7 ± 7.7^a^	75.8 ± 7.6^b^	63.1 ± 8.2	0.00012^**^
Gender (Male/Female)	8/6	6/6	7/9	0.74
IQ	114 ± 16.9	118 ± 8.1	115 ± 9.4	0.68
NART	12.2 ± 10.3	10.7 ± 7.1	12.6 ± 7.5	0.83
MMSE	23.0 ± 3.6^a^	28.0 ± 1.5	28.7 ± 1.7	<0.0001^**^
Rey Copy	25.6 ± 10.8	32.2 ± 4.3	34.5 ± 2.8	0.022^*^
Rey Imm	5.2 ± 4.6^a^	9.8 ± 6.3	18.2 ± 11.5	0.004^*^
Rey Del	3.2 ± 4.2^a^	10.5 ± 6.6	18.0 ± 7.5	<0.0001^**^
WLM Imm	15.4 ± 9.8^a^	30.0 ± 10.5^b^	45.2 ± 8.5	<0.0001^**^
WLM Del	3.8 ± 6.3^a^	13.1 ± 8.2^b^	28.4 ± 6.2	<0.0001^**^
Hopkins Imm	9.8 ± 8.8^a^	18.3 ± 6.8^b^	26.2 ± 8.6	0.00016^**^
Hopkins Del	1.6 ± 1.9^a^	6.7 ± 9.1^b^	12.4 ± 7.1	0.003^*^
Hopkins RI	5.0 ± 3.8^a^	7.9 ± 3.9^b^	11.4 ± 0.9	<0.0001^**^
LNS	3.6 ± 2.4^a^	6.3 ± 3.0^a^	10.71 ± 3.20	<0.0001^**^
Semantic Fluency	11.3 ± 3.2^a^	14.7 ± 4.2^b^	23.8 ± 8.6	<0.0001^**^
Verbal Fluency	35.6 ± 11.9^a^	38.7 ± 13.5^b^	49.2 ± 12.9	0.038^*^
HADS Anxiety	6.8 ± 3.8	6.0 ± 3.9	5.2 ± 3.5	0.58
HADS Depression	5.6 ± 4.3	4.0 ± 2.4	3.2 ± 3.6	0.28

*SD* standard deviation, *NART* National Adult Reading Test, *MMSE* Mini-Mental State Examination, *Rey* Rey–Osterrieth complex figure test, *Imm* immediate recall, *Del* delayed recall, *WLM* Wechsler Memory Scale–Logical Memory test, *Hopkins* Hopkins Verbal Learning Test, *RI* Recognition Index, *LNS* Letter-Number Sequencing, *HADS* Hospital Anxiety and Depression Scale.

*p  < 0.05; **p < 0.001 in ANOVA.

^a^Significant difference between Alzheimer’s dementia patients and healthy controls in post hoc comparisons (*p* < 0.05).

^b^Significant difference between mild cognitive impairment patients and healthy controls in post hoc comparisons (*p* < 0.05).

### ^11^C-PBR28 PET

**Fig. 1:**
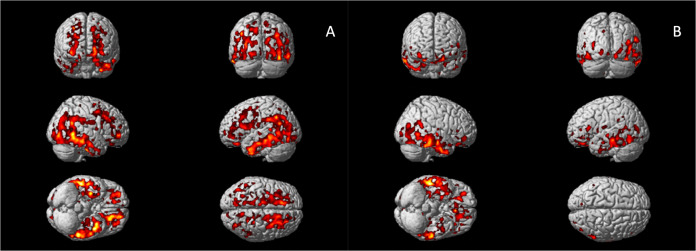
Voxel-wise group comparisons of ^11^C-PBR28 PET images. **A** Increased ^11^C-PBR28 uptake ratio in the Alzheimer’s dementia patients compared to healthy controls; (**B**) Increased ^11^C-PBR28 uptake ratio in the Aβ+ mild cognitive impairment patients compared to healthy controls.

Voxel-wise analysis confirmed increased ^11^C-PBR28 uptake in bilateral superior and middle frontal gyri, bilateral frontal pole, bilateral middle and inferior temporal gyri, bilateral fusiform gyri, bilateral lateral occipital gyri, left hippocampus, left parahippocampal gyrus, and left angular gyrus in AD patients (corrected for age and gender, Fig. 1A). In Aβ+ MCI patients, elevated ^11^C-PBR28 uptake was seen in right inferior and middle temporal gyri, right temporal pole, right fusiform gyrus and right parahippocampal gyrus (corrected for age and gender, Fig. 1B). ROI analysis localised elevated ^11^C-PBR28 uptake in medial temporal lobe in AD patients compared to HCs (1.09 ± 0.11 versus 1.01 ± 0.07, *p* = 0.029). No significant difference was found between the MCI patients (1.04 ± 0.08) and HCs. TSPO genotypes did not show significant influence on SUVR values at both ROI level and voxel level.

### Cross-group comparisons of network metrics and cortical thickness

**Fig. 2:**
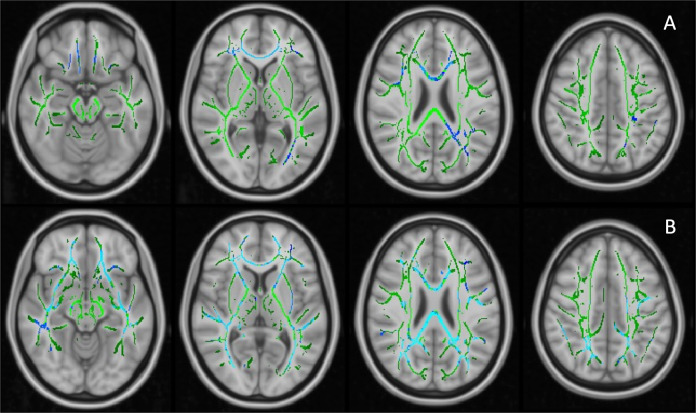
Voxel-wise analysis of FA values on TBSS skeletons. **A** Decreased FA values in Alzheimer’s dementia patients compared to healthy controls, age and gender adjusted; (**B**) Significant negative relationship between ^11^C-PBR28 uptake (^*11*^*C-PBR28 PC1*) and FA values in Aβ+ patients, corrected for age, gender, cortical thickness and ^18^F-flutemetamol uptake. Significant clusters (FDR corrected) are shown in blue (negative association); TBSS skeleton is shown in green.

The first PC of FA values (*PC1*, 40.3% variance explained) did not differ across the groups, while *PC2* (9.7% variance explained) of the AD patients was significantly lower than HCs (*p* = 0.003, age and gender corrected). The *FA PC2* axis distinguished AD and HCs with good separation (Supplementary Fig. 2). Loading weights revealed that all TOIs had positive weights on *FA PC1* while long association fibres had higher weights on *PC2*, suggesting *FA PC1* as measure of overall white-matter integrity and *FA PC2* as a marker of association fibres, where disease-specific changes might happen (Supplementary Fig. 2). Voxel-wise comparison showed decreased FA in the right inferior longitudinal fasciculus (ILF) and right superior longitudinal fasciculus (SLF) in the AD patients compared to HCs (corrected for age and gender, Fig. 2A).

**Fig. 3:**
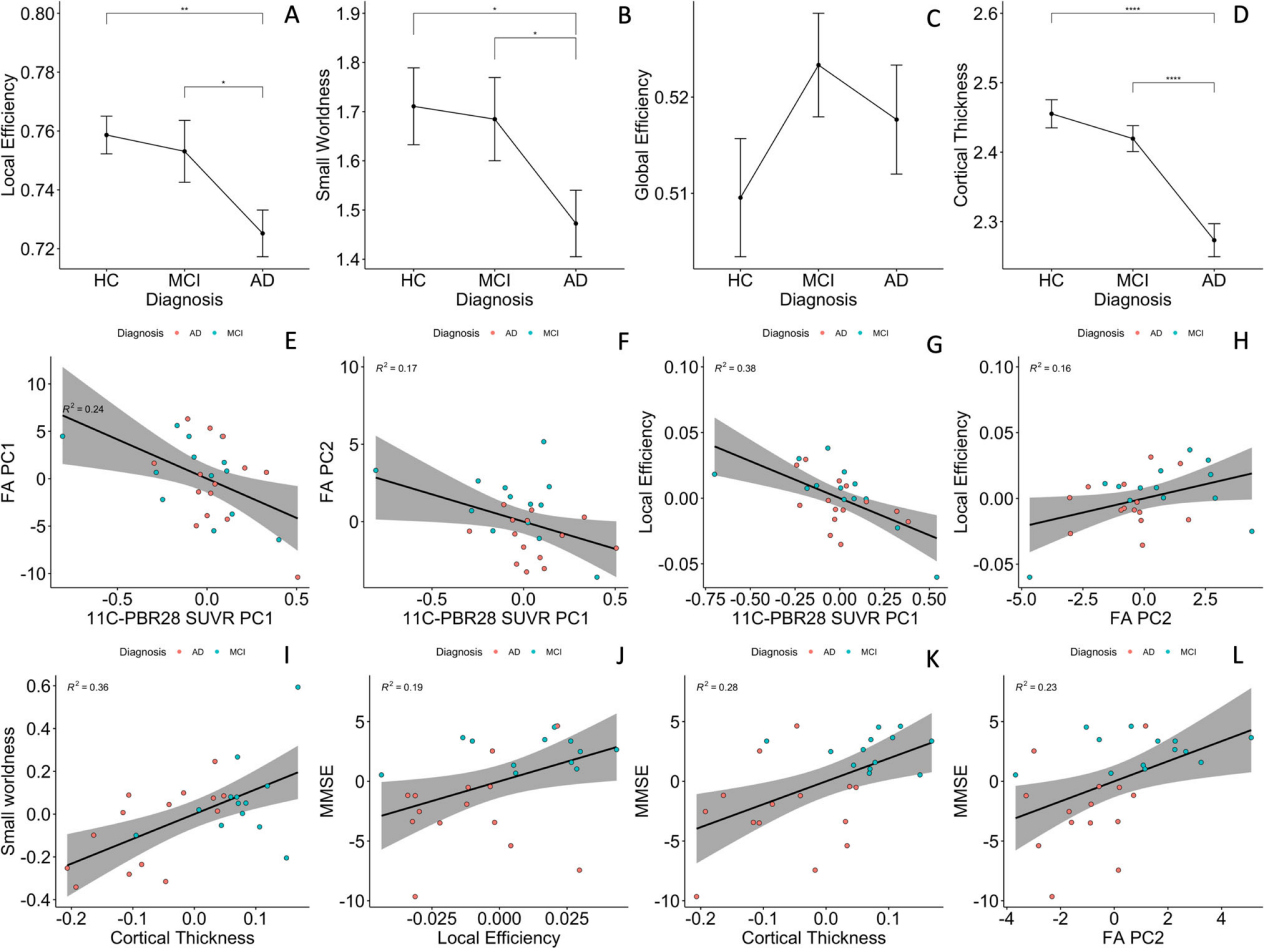
Group comparisons and partial correlations. **A–C** Group comparisons of functional network topology metrics found reduced small-worldness and local efficiency in patients with Alzheimer’s dementia compared to healthy controls; (**D**) patients with Alzheimer’s disease had significantly lower cortical thickness compared to healthy controls and mild cognitive impairment participants. **E**, **F** Cortical ^11^C-PBR28 uptake has significant negative correlations with PC1 and PC2 of FA values in Aβ+ patients; (**G**) cortical ^11^C-PBR28 uptake has significant negative correlation with local efficiency; (**H**) FA PC2 positively correlates with local efficiency; (**I**) cortical thickness positively correlates with small-worldness; (**J–L**) FA PC2, local efficiency and cortical thickness positively correlate with mini-mental state examination scores. AD Alzheimer’s dementia, MCI mild cognitive impairment, HC healthy controls, PC principal component, FA fractional anisotropy. *p < 0.05 in pairwise comparisons; ****p < 0.0001 in pairwise comparisons.

Functional network small-worldness (SW) and local efficiency (Eloc) were significantly decreased in the AD patients, while global efficiency (Eg) was not (1.47 ± 0.25 versus 1.71 ± 0.31, 0.72 ± 0.03 versus 0.75 ± 0.02, and 0.52 ± 0.02 versus 0.51 ± 0.02, *p* = 0.029, 0.003, and 0.34, respectively Fig. 3A–C). AUC of SW and Eloc were also lower in AD patients (0.69 ± 0.08 versus 0.74 ± 0.09, and 0.339 ± 0.005 versus 0.343 ± 0.004, *p* = 0.062 and 0.074, respectively, Supplementary Fig. 3A–C).

AD patients had decreased average cortical thickness (2.27 ± 0.09 mm) compared to HCs (2.46 ± 0.08 mm, *p* < 0.0001), but MCI patients (2.42 ± 0.07 mm) did not show significant cortical thinning (*p* = 0.21, Fig. 3D).

### Influence of cortical neuroinflammation and amyloid deposition on network structural integrity in the Alzheimer’s continuum

In Aβ+ patients, cortical thickness had a positive association with *FA PC2* (standardised *β* = 0.48, 95% CI 1.37–0.85, *p-perm* = 0.027, corrected for age and gender), while ^*18*^*F-flutematemol PC1* was not associated with either of the *FA PC*s, suggesting that association fibre integrity is quantitatively associated with grey-matter preservation, but not Aβ plaque load. ^*11*^*C-PBR28 PC1* was negatively associated with both *FA PC1* and *FA PC2* (standardised *β* = −0.44, 95% CI: 0.11–0.13, *p-perm* = 0.018; standardised *β* = −0.375, 95%CI: −0.75 to −0.01, *p-perm* = 0.037, corrected for cortical thickness in addition to age and gender, Fig. 3E, F). To test whether microglial activation had stage-dependent relationship with brain structural network integrity at MCI and AD stages, we also performed same linear analysis in the two groups separately, and the results suggested same direction of association (Supplementary Materials Fig. 6).

To further explore the influence of inflammation and Aβ in different cortices on structural network integrity, SCCA was performed between PET and FA measures Negative correlation was found between ^11^C-PBR28 and FA values (*λ* = 0.70, *r* = 0.62, *p-perm* = 0.034), with ^11^C-PBR28 uptake in bilateral frontal, parietal and posterior cingulate cortices having non-zero weights. Canonical correlation between ^18^F-flutematemol and FA was not significant (*λ* = 0.50, *r* = 0.43, *p-perm* = 0.53), reinforcing the above findings that neuroinflammation, but not amount of Aβ in the cortex is associated with white-matter damage in Alzheimer’s disease. Interestingly, when both ^11^C-PBR28 and ^18^F-flutematemol were included, ^18^F-flutematemol uptake information did not improve the model fits under different level of sparsity constraints (Supplementary Fig. 5). Under heavy LASSO regularisation (*λ* < 0.50), none of the ^18^F-flutematemol ROIs was assigned non-zero weight. Even under light regularisation, the ^18^F-flutematemol ROIs had minimal canonical weights in the model (Table 2), and the overall model performance was less satisfactory compared with the model with ^11^C-PBR28 only (*λ* = 0.70, *r* = 0.60, *p-perm* = 0.064).

**Table 2:** Canonical correlation between microglial activation/amyloid deposition and FA values.

			Canonical weights			
	PET ROIs	PET	FA	PET	FA	FA TOIs
Flute	Frontal/L	–	0.16	−0.04	0.15	Corpus callosum Genu
	Frontal/R	–	0.51	−0.01	0.48	Corpus callosum body
	Temporal/L	–	0.61	0	0.60	Corpus callosum splenium
	Temporal/R	–	0.163	0	0.06	fornix
	Parietal/L	–	0.09	−0.04	0.10	ILF&IFOF R
	Parietal/R	–	0.24	−0.05	0.25	ILF&IFOF L
	Occipital/L	–	0.21	0	0.22	Cingulum (cingulate) R
	Occipital/R	–	0.31	0	0.29	Cingulum (cingulate) L
	Ant. Cing.	–	0	−0.03	0	Cingulum (hippocampus) R
	Post. Cing.	–	0	−0.04	0	Cingulum (hippocampus) L
PBR	Frontal/L	−0.44	0.12	−0.38	0.05	Stria terminalis R
	Frontal/R	−0.42	0.15	−0.38	0.05	Stria terminalis L
	Temporal/L	0	0.06	−0.22	0.07	SLF R
	Temporal/R	0	0	0	0	SLF L
	Parietal/L	−0.55	0	−0.41	0	SFOF R
	Parietal/R	−0.34	0	−0.38	0	SFOF L
	Occipital/L	0	0.10	−0.22	0.16	UF R
	Occipital/R	0	0.10	−0.20	0.15	UF L
	Ant. Cing.	0	0.16	−0.31	0.26	Tapetum R
	Post. Cing.	−0.46	0.15	−0.38	0.24	Tapetum L

Canonical weights in correlation between PET tracer uptakes and FA values in SCCA analysis. The first two columns show canonical correlation between ^11^C-PBR28 PET SUVR and FA values; the last two columns show canonical correlation between ^11^C-PBR28, ^18^F-flutematemol SUVR and FA values. The lasso penalty (λ) was 0.70 in both SCCA analysis presented in the table. Negative weights on tracer uptake measures and positive weights on FA values indicate negative relationship between the two set of variables.

*ILF* inferior longitudinal fasciculus, *IFOF* inferior fronto-occipital fasciculus, *SLF* superior longitudinal fasciculus, *SFOF* superior fronto-occipital fasciculus, *UF* uncinate fasciculus, *L* left, *R* right, *Ant* anterior, *Post* posterior, *Cing* cingulate, *Flute* ^18^F-flutemetamol regional uptake, *PBR* ^11^C-PBR28 regional uptake.

A voxel-wise regression analysis, which included age, gender, cortical thickness, ^18^F-flutemetamol and ^11^C-PBR28 uptake, supported the SCCA findings. ^*11*^*C-PBR28 PC1* was associated with decreased FA in the forceps major, right inferior fronto-occipital fasciculus (IFOF) and ILF in Aβ+ patients (Fig. 2B).

Altogether, the above findings suggested a negative influence of cortical neuroinflammation on brain structural network integrity, which is independent of grey-matter atrophy and Aβ deposition in AD.

### Influence of cortical neuroinflammation and amyloid deposition on network functional organisation in the Alzheimer’s continuum

In the Aβ+ patients, ^*11*^*C-PBR28 PC1* negatively correlated with local efficiency (standardised *β* = −0.468, 95% CI: −0.846 to −0.018, *p-perm* < 0.001, cortical thickness corrected in addition to age and gender, Fig. 3G), but not global efficiency nor small-worldness. AUC of network Eloc was also negatively associated with ^11^C-PBR28 PC (standardised *β* = −0.432, 95% CI: −0.775 to −0.160, *p-perm* = 0.045). ^18^F-flutematemol PC did not correlate with the network topology metrics. Similar results was found in AD and MCI groups in sub-group analyses (Supplementary Fig. 6). The above findings suggest that cortical neuroinflammation is associated with disruption of brain functional network organisation, in particular the effective processing of information within locally interconnected subnetworks.

### Network structural integrity, functional network organisation and cognition

*FA PC2* had significant positive association with local efficiency (standardised *β* = 0.44, 95% CI: 0.08–0.79, *p-perm* = 0.047, cortical thickness corrected in addition to age and gender, Fig. 3H), while cortical thickness correlated with small-worldness (standardised *β* = 0.61, 95% CI: 0.21–1.01, *p-perm* = 0.001, PC2 corrected in addition to age and gender, Fig. 3I). Similar results were obtained using Eloc and SW AUC in regression analysis (standardised *β* = 0.45, 95%CI: 0.05–0.85 *p-perm* = 0.021 and standardised *β* = 0.56, 95% CI: 0.16–0.96 *p-perm* = 0.005, Supplementary Fig. 3). These findings suggested that effective functional brain network organisation is dependent on both grey-matter and structural network integrity.

*FA PC2*, local efficiency and cortical thickness positively correlated with MMSE (standardised *β* = 0.44, 95%CI: 0.04–0.84, *p-perm* = 0.025; standardised *β* = 0.52, 95% CI: 0.22~0.82, *p-perm* = 0.031; and standardised *β* = 0.60, 95% CI: 0.29–0.92, *p-perm* = 0.0004, respectively, Fig. 3J–L). Local efficiency AUC also marginally correlated with MMSE (standardised *β* = 0.39, 95% CI: −0.007–0.79, *p-perm* = 0.052, Supplementary Fig. 3). The above findings supported the idea that brain structural network integrity, effective functional network organisation and grey-matter preservation are the basis of cognition.

As there was a potential outlier in the above association analyses (Fig. 3), we have identified the outlier (a 75-year-old MCI patient) and re-examined the above findings. The results remain similar to the original ones (Supplementary Material 9, Supplementary Fig. 7), supporting the above inferences.

We have also examined whether age have confounded the previous findings. We found that in both healthy volunteers and the combined cohort (diagnostic groups considered), age did not have significant effect on the metrics we used in the study (probably due to moderate sample size). To further ensure the above inferences were not confounded by age, we have also performed a 5:5:5 exact match of HC, MCI and AD patients by age (within 1 year), and observed similar results (Supplementary Material 10).

### Direct effect and indirect effect of neuroinflammation on functional network

As neuroinflammation was associated with both network structural integrity and functional organisation, we evaluated whether the effect of neuroinflammation on functional network was mediated by structural integrity. A linear mediation analysis with ^11^C-PBR28 and ^18^F-flutematemol PCs, *FA PC2* and age as independent variables, Eloc as dependent variable and *FA PC2* as mediator showed a significant average direct effect of ^*11*^*C-PBR28 PC* on local efficiency (95% quasi-Bayesian credible interval −0.25 to −0.06, *p* = 0.002) and a marginal average causal mediation effect via *FA PC2* (95% quasi-Bayesian credible interval −0.12 to −0.001, *p* = 0.066). The above analysis suggested that neuroinflammation may have both direct and indirect negative influence (via structural network damage) on brain functional organisation disruption.

## Discussion

This study evaluated the influence of neuroinflammation and Aβ deposition on brain structural and functional network in Alzheimer’s disease using a cross-sectional multimodal imaging dataset. Our findings suggest that neuroinflammation, but not Aβ deposition, directly influences the effective functioning of brain networks, which in turn is responsible for cognitive impairment in AD. By adjusting for amyloid deposition in the linear model, we were able to illustrate that microglial activation had a unique contribution to (putatively subsequent) network disruption, which is not dependent on the quantity of amyloid deposition. The current findings have bridged our understanding of macroscopic brain connectivity change and microscopical pathological processes in AD, and suggested a pathway from neuroinflammation to cognitive impairment via network disruption.

Neuroinflammation has been established as an important component in Alzheimer’s pathology [26]. Neuroimaging studies have found that microglial activation is associated with lower baseline cognition and faster cognitive decline [27, 28]. At early MCI stage, neuroinflammation, but not NFT, was found to correlate with Aβ deposition [29], while in AD patients tau pathology was more related to neuroinflammation [30], suggesting a possible mediation role of neuroinflammation. This directional flow from neuroinflammation to tau aggregation is further supported by post-mortem pathological and genetic analyses [31].

Alzheimer’s dementia is increasingly understood as a consequence of brain network dysfunction. rs-fMRI studies have reported impaired network connectivity and abnormal network topology in AD and MCI patients [32–36]. In our study, we confirmed the presence of significantly decreased small-worldness and network segregation (Eloc) in the AD patients, implying a disrupted brain functional network for information processing.

The basis of the functional network is inevitably structural network [37], and white-matter degeneration is an early feature of AD [9]. We found decreased microstructural integrity in association fibres in AD patients, which correlated with cognition, consistent with the literature [38]. More interestlingly, Aβ and hyperphosphorylated tau may propagate in a prion-like manner via the white-matter tracts [39, 40], highlighting the important position of brain structural and functional connectivity in AD pathegenesis.

Recently, it has been reported that anterior temporal neuroinflammation is associated with longitudinal cognitive decline in Aβ+ patients [28], and that an independent component of brain neuroinflammation is associated with abnormal functional connectivity within and between brain networks [41]. Given the current evidence, we further investigated the influence of neuroinflammation on brain structural and functional network’s overall organisation. We have demonstrated that neuroinflammation is an independent predictor of structural and functional network disruption, while the amout of Aβ in Aβ+ population was not linearly associated with brain connectivity changes. On the one hand, the lack of linear association between amyloid deposition and structural/functional network disruption might reflect non-linear relationships [42]. One the other hand, it may suggest that once the disease process has been initiated, the quantity of Aβ deposition no longer directly influcences brain network and cognitive decline [1]. Indeed, this hypothesis could explain the controversial results from clinical trials targeting Aβ in the symptomatic stage of Alzheimer’s disease [43].

During brain development, microglia contribute to neurogenesis, axon outgrowth, synapse modelling and myelination [44]. In adulthood, microglia have dynamic and direct interactions with neuronal network structures [45] and are involved in the maintenance and repair of myelin [44]. In AD brains, activated microglia undergo phenotypic changes and migrate to Aβ plaques and NFTs [46]. Activated microglia can cause direct damage to oligodendrocytes and axons through ROS production (reactive oxygen species), matrix metalloproteinases and pro-inflammatory cytokines [47]. Accumulation of activated microglia has also been reported to co-locate with damaged axonal initial segments in a mouse model of AD [48]. Furthermore, an ex vivo experiment has demonstrated that activated microglia can induce neuritic beading of axons, inhibit mitochondrial function and axonal transport through N-methyl-D-aspartate (NMDA) receptor signalling [49]. The preclinical evidence above explains the biological basis of how neuroinflammation could induce structural and functional network disruption.

**Fig. 4:**
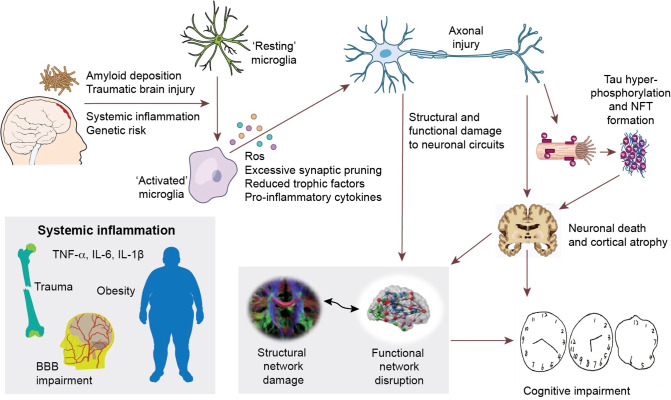
Hypothetical framework of pathological events leading to structural and functional network impairment and cognitive decline in Alzheimer’s disease. The initial amyloid deposition, together with other inflammatory risk factors (including systemic inflammation, traumatic brain injuries, and genetic risk factors, etc.) initiate neuroinflammation, which could cause neuronal damage via production of reactive oxygen species, pro-inflammatory cytokines, reduced trophic support and direct synaptic pruning. These aforementioned events could then directly damage neuronal circuits and cause abnormal neuronal activity, resulting in disruption of structural and functional brain connectivity. On the other hand, these events could also facilitate the propagation of hyperphosphorylated tau species and formation of intracellular neurofibrillary tangles (NFT). NFT formation further causes neuronal dysfunction, axonal destabilisation and neuronal death, hence cortical atrophy and network dysfunction. This cascade of events eventually leads to cognitive impairment in Alzheimer’s disease.

Our findings, together with previous evidence, suggests a framework of systemic brain damage in Alzheimer’s continumm, where Aβ deposition initiates neuroinflammation, which then directly lead to structural and functional brain network damage, while also promoting hyperphosphorylated tau formation and aggregation. The tau pathology, in turn, is associated with further network damage as well as cortical atrophy [10, 50]. Structural network and functional network disruption, as well as cortical atrophy, eventually result in cognitive impairment (Fig. 4). It is possible that the initial activation of microglia by other inflammatory factors such as trauma, infection and systemic inflammation, contributes to future impairment of brain connectivity, formation of tau pathology, and progression of the disease [4, 51]. Recently, a ground breaking multicentre study by Pascoal et al. have demonstrated that microglial activation may propagate together with tau pathology in AD, suggesting that the prion-like network-based tau propagation is associated with basline neuroinflammatory network [51]. Together with previous evidence, it is worth considering whether microglial activation at downstream regions, caused by amyloid deposition and various pathological factors, could pave the way for the prion-like propagation of tau protein or local dissemination of tau pathology.

While there is evidence that at the very early stage of the disease, a stronger response of anti-inflammatory microglia is associated with better outcome [52, 53], in the later stages, the detection of β-sheeted fibrillary amyloid by PET, might already mark the failure of the protective response, followed by a deleterious inflammatory response.

The limitations of the current study are: (1) its cross-sectional nature and thus causal relationships need validation by longitudinal studies; (2) The age difference between healthy volunteers and cognitively impaired patients were rather large, and may have influenced the cross-group comparisons, dispite the efforts made to correct for the matter using linear regression mothods. The mismatch of age could be due to the fact that healthy volunteers were recruited early on in the current study, and a more balanced recruitment is recommended for future studies. (3) The hypothetical framework between neuroinflammation, tau aggregation and downstream network damage and cortical atrophy also needs to be further tested, especially the how inflammation may interact with the tau propagation and how the two factors may influence brain network together.

In conclusion, our findings reinforce the theory that a network failure underlies cognitive impairment in AD, and is compatible with the framework in which neuroinflammation, initiated by amyloid pathology, plays a significant upstream role in causing structural and functional connectivity impairment as the disease progresses. Therefore, it is essential to consider: (1) the inclusion of neuroinflammation in the current ‘amyloid-tau-neurodegeneration’ framework and (2) targeting neuroinflammation in the symptomatic stage of Alzheimer’s disease to preserve brain networks and delay disease progression.

## Supplementary information

Supplementary materials

## Data Availability

The authors confirm that the data supporting the findings of this study are available within the article and its Supplementary Material. Data that support the findings of this study are available from the corresponding author, upon reasonable request.
