# Supplementary material for: Neuroinflammation is independently associated with brain network dysfunction in Alzheimer’s disease
Source: Mol Psychiatry. 2022 Dec 6;28(3):1303–11. doi: 10.1038/s41380-022-01878-z (PMC10005956; doi:10.1038/s41380-022-01878-z)
Supplement: Supplementary file 1 — Supplementary materials [file 41380_2022_1878_MOESM1_ESM.docx]

**Supplementary materials**

**Supplementary file 1: Methodological considerations on network sparsity threshold**

To avoid bias associated with a specific sparsity threshold, we computed the network small-worldness, global efficiency and local efficiency under a range of sparsity thresholds (from 0.05 to 0.50). We observed that after the threshold of 0.20 was reached, the network topology metrics tend to stabilise. The area under curve (AUC) of each network metric under network sparsity threshold from 0.05 to 0.50 were calculated and were used to confirm the findings.


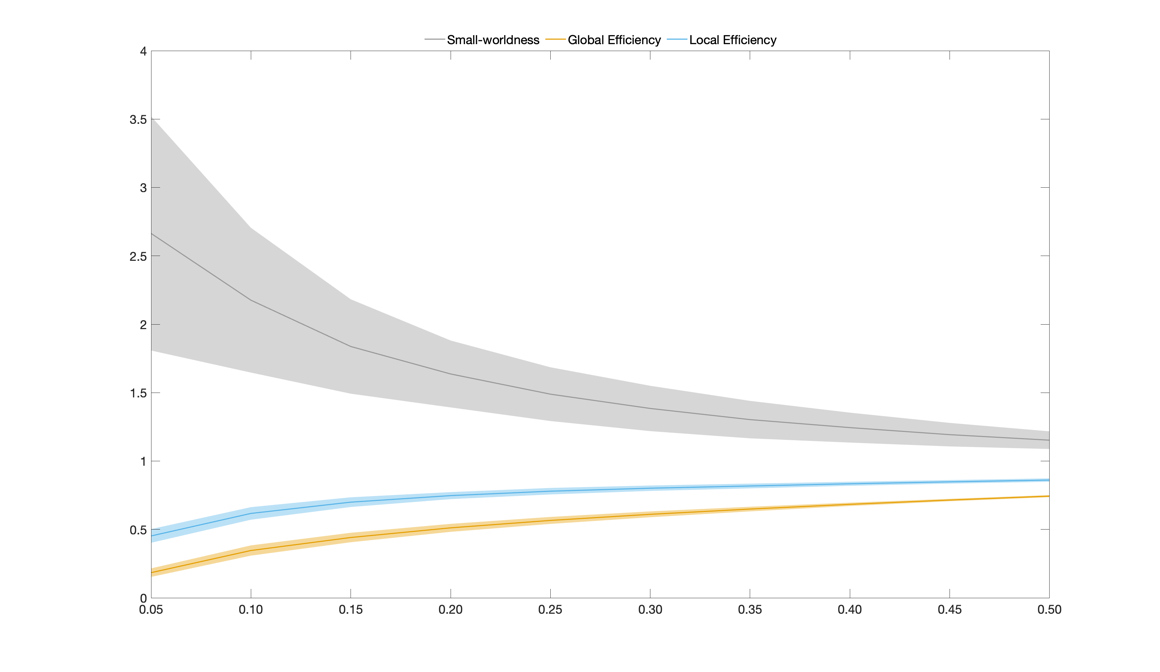


**Supplementary Figure 1. Mean values of network topology metrics under different sparsity thresholds of functional connectivity matrix.**

Solid lines show the mean value of the whole study cohort and shaded area show the standard deviation of values. X-axis is value of sparsity threshold; Y-axis is the value of network metrics.

Network segregation refers to the ability of the brain to process information within an interconnected subgroup of regions. These subgroups of nodes may be specialised for certain tasks and, using several segregated subgroups, the brain can process various tasks simultaneously with high efficiency. Network integration, on the other hand, reflects the brain network’s ability to summarise or combine information across segregated regions. In other words, it measures how easy it is for information to be passed from a node to any other node in the brain. This graph can be represented by the average shortest path length (edges that must be passed) from node A to any other node in the network^1^. A cost-efficient brain network must balance the need to have both high local efficiency and high global efficiency, and it has been established that the brain uses a small-world organisation to achieve the balance. Technically, small-world networks are those which have approximately the same level of network integration as random networks, but still have significantly higher level of network segregation compared to random networks^2^. This is achieved by having highly connected hubs in the network, the edges between which form common pathways for connections between members of different clusters. The metric small-worldness is coined to reflect how effectively the network is organised in a small-world fashion and is expressed as the ratio between network G’s clustering coefficient and clustering coefficient of similar random networks, divided by the ratio between G’s characteristic path length and that of similar random networks^3^.

The exact formulae for computation are as follow:

$$E_{g}=\frac{1}{n}\sum_{i\in N} E_{i}=\frac{1}{n}\sum_{i\in N} \frac{\sum_{j\in N.j\neq i} d_{ij}^{-1}}{n-1}$$

$$E_{loc}=\frac{1}{n}\sum_{i\in N} E_{loc,i}=\frac{1}{n}\sum_{i\in N} \frac{\sum_{j,h\in N.j\neq i} {a_{ij}a_{ih}[d_{jh}\left( N_{i} \right)]}^{-1}}{k_{i}(k_{i}-1)}$$

$$SW=\frac{C/C_{rand}}{L/L_{rand}}$$

Where *E_g_* is the global efficiency, *E_loc_* is the network local efficiency and *SW* is the small-worldness. *E_i_* is the efficiency of node *i*, *d_ij_* is the shortest path between node *i* and *j.* *E_loc,i_* is the local efficiency of node *i*, *d_jh_(N_i_)* is the shortest path between two neighbour nodes of *i*, (*j* and *h),* and *K_i_* is the degree of node *i*, which is the number of edges connected to the node. *C* and *C_rand_* are clustering coefficients, *L* and *L_rand_* are characteristic path lengths of current network and random networks.

**Supplementary file 2: Factor loadings of FA principal components**

Principal component analysis was performed using FA values sampled from 48 tracts of interest (from DTI-81 white matter label atlas). The first principal component of FA values (PC1) explained 40.3% variance of data, while PC2 explained 9.7% of variance. Principal component 2 axis distinguishes AD and healthy participants with relatively good separation, with healthy controls having positive PC2s and AD patients having negative values.

Loading weights of TOIs revealed that all TOIs had positive weights on PC1 while there were differential loadings on PC2, with long association fibres having mostly positive weights (Supplementary Fig.2). It is probable that the first component represents the overall integrity of white matter structural network, while the second component reflect a process in which association fibres and projection fibres are influenced differently, likely due to Alzheimer’s disease.


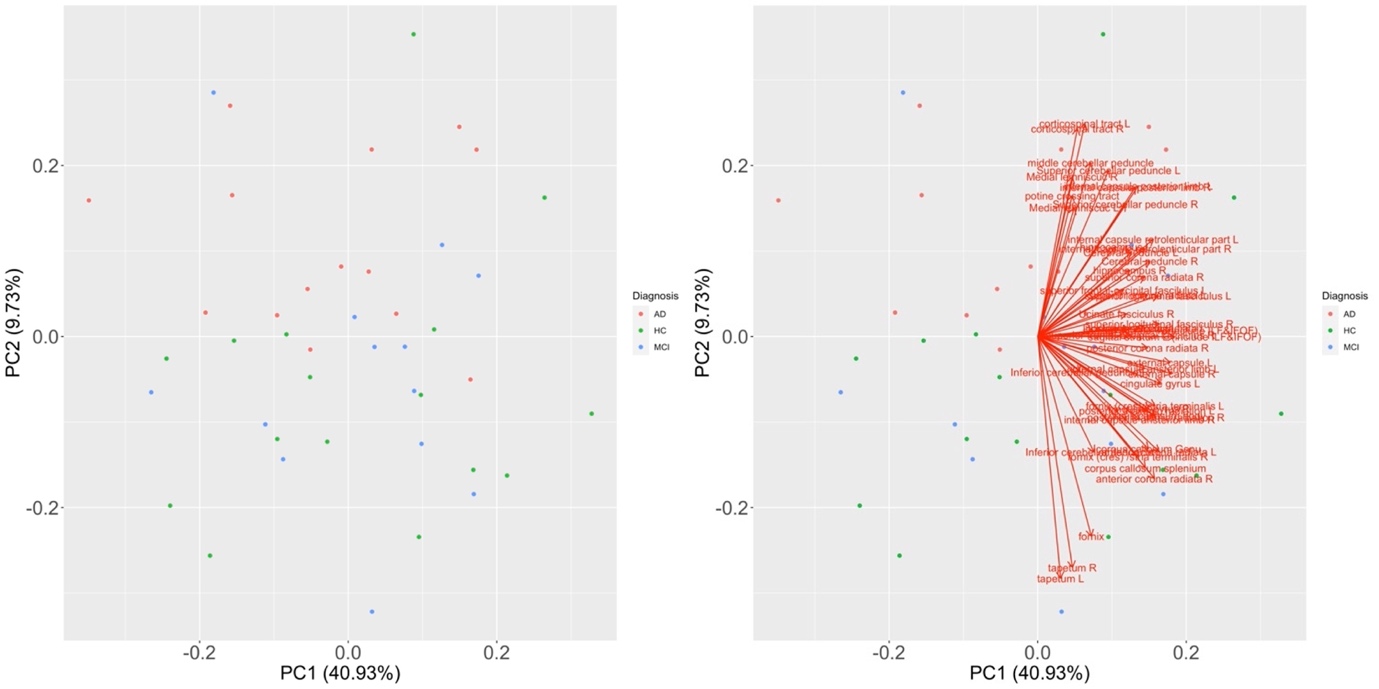


**Supplementary Figure 2. Factor loadings of tracts of interest on principal components of FA values.**

**Supplementary file 3: Factor Loadings in PCA analysis on PET measures**

PCA was also applied to ^11^C-PBR28 and ^18^F-flutemetamol uptakes of major cortices. The loadings of each ROI are summarised in supplementary table 1. ^11^C-PBR28 PC1 explained 78.9% of total variance in ^11^C-PBR28 ROI data; ^18^F-flutemetamol PC1 explained 96% of total variance in ^18^F-flutemetamol data.

Supplementary Table 1. Factor Loadings in PCA analysis on PET measures

|  | Loadings on ^11^C-PBR28 PC1 | Loadings on ^18^F-Flutemetamol PC1 |
| --- | --- | --- |
| Frontal Lobe L | 0.34 | 0.33 |
| Frontal Lobe R | 0.32 | 0.34 |
| Temporal Lobe L | 0.16 | 0.27 |
| Temporal Lobe R | 0.12 | 0.28 |
| Parietal Lobe L | 0.36 | 0.33 |
| Parietal Lobe R | 0.36 | 0.3 |
| Occipital Lobe L | 0.23 | 0.21 |
| Occipital Lobe R | 0.21 | 0.25 |
| Anterior Cingulate | 0.38 | 0.4 |
| Posterior Cingulate | 0.49 | 0.4 |

**Supplementary file 4: Details of sparse canonical correlation analysis**

Whereas canonical correlation analysis seeks vectors ***u*** and ***v*** (canonical variates) that maximise correlation between two sets of observations of variables, the SCCA analysis imposes lasso constraints to the maximisation problem to ensure sparsity of the solution and prevent overfitting problems. Specifically, the lasso penalty limits the L1-norm of each canonical variate within the penalty term λ (ranging from 0 to 1, with larger λ having less constraint) ^4^. In the current study, a range of λ (from 0.10 to 0.70 incrementing at 2/3) was tested and the model fits (Z-statistics) were inspected. For simplicity, the statistical test results (canonical correlation coefficient and p-values) and canonical weights of individual variables were presented with the λ that gave best model fit. 5000 permutations were performed to determine the significance of canonical correlation. Only the first canonical variate is considered in the current analysis.

Canonical correlation analysis (CCA) was originally developed by Hotelling (1936). Given two sets of variables **X** (*n* by *p* matrix) and **Y** (*n* by *q* matrix), with *n* denoting number of observations, *p* and *q* denoting number of variables. CCA finds eigenvectors **u** and **v** (canonical variates) that maximise the function Cor(**Xu**, **Yv**):

$${maximise}_{u,v}\left( u^{T}X^{T}Yv \right) subject to u^{T}X^{T}Xu=1 and v^{T}Y^{T}Yv=1$$

This maximisation problem gives the solution for **u** and **v**, which is not sparse, and are not unique if *p* and *q* are larger than *n*. The non-sparsity of solution could sometime cause difficulty in interpreting the results especially when *p* and *q* are large, for example, the difficultly to identify the most relevant variables as all variables are assigned canonical weights. The maximisation without penalty could also lead to the concern of overfitting the data. Witten et al. proposed to add a lasso penalty to the maximisation problem, i.e., limiting the L1-norm of vector **u** and **v** within certain λ (0< λ$\leq$1), rendering the maximation problem under regularisation:

${maximise}_{u,v}\left( u^{T}X^{T}Yv \right) subject to u^{T}X^{T}Xu\leq1, v^{T}Y^{T}Yv\leq1, \left| \left| u \right| \right|_{1}\leq\lambda, \left| \left| v \right| \right|_{1}\leq\lambda$

This will give a sparse solution for **u** and **v**, which alleviate the above problems in CCA.

**Supplementary file 5: Analysis with AUC of Network Topology Metrics**

Analysis with AUC of functional network topology metrics yielded similar results as those using exact metrics at sparsity threshold 0.2, although the AUCs cross the range of sparsity thresholds had less sensitivity in comparison.


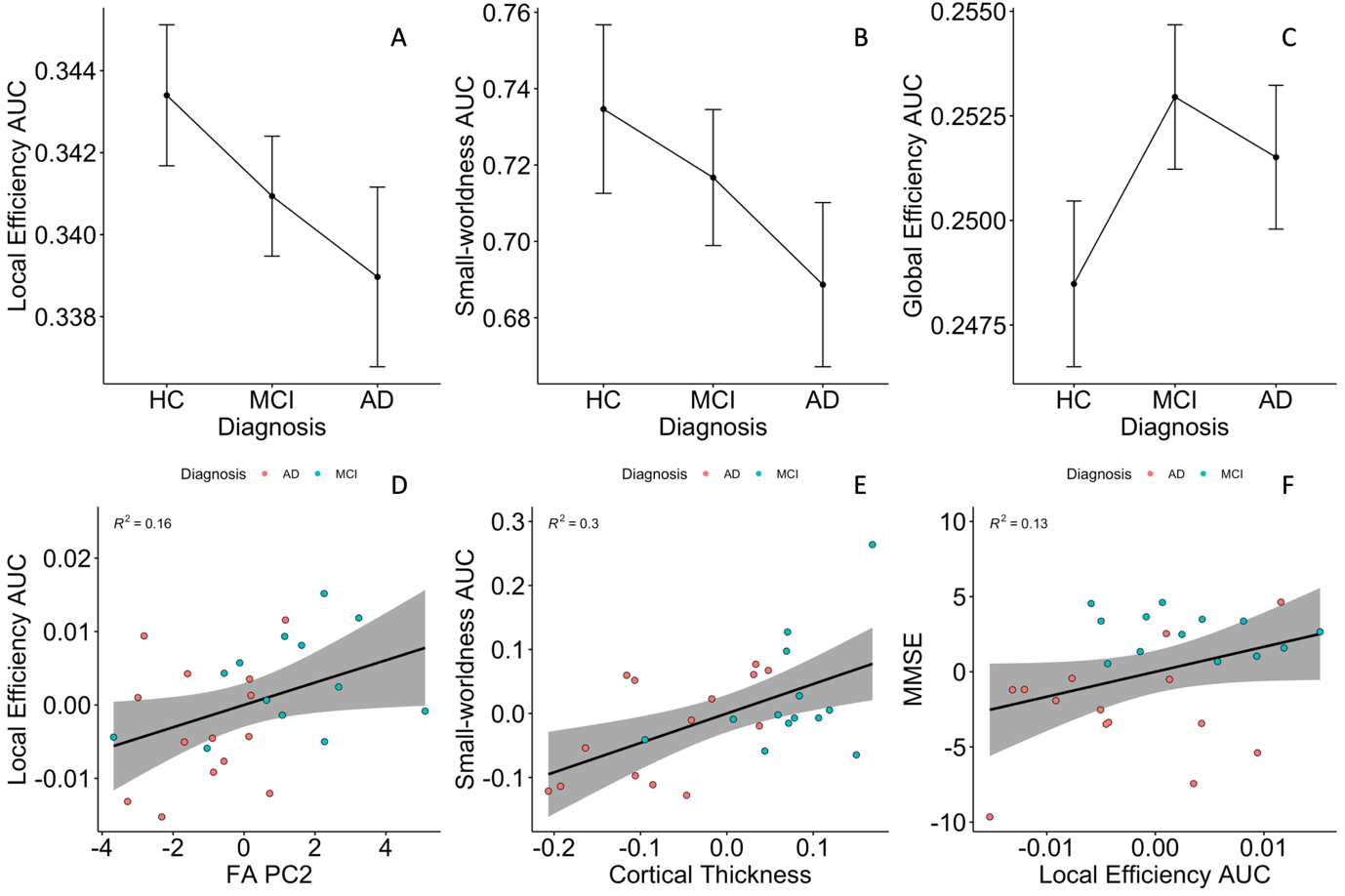


**Supplementary Figure 3. Analysis with AUC of Network Topology Metrics.** **(A-C)** Group-wise comparisons of network topology AUCs. **(D-F)** Regression analyses using AUCs yielded similar results as analyses using exact values. AD: Alzheimer’s Dementia; HC: Healthy Controls; MCI: Mild Cognitive Impairment; AUC: Area Under Curve; FA: Fractional Anisotropy; PC: Principal Component.

**Supplementary file 6: Partial correlations between ROIs of ^11^C-PBR28 and FA values in tracts of interest in Aβ+ patients**

To explore the relationship between neuroinflammation and white matter network integrity, the correlation between FA values and ^11^C-PBR28 uptake across multiple regions was explored. Negative relationship between the two variables was found in multiple ROI-TOI pairs in both mild cognitive impairment and Alzheimer’s dementia patients. A graphic representation of the correlation in Aβ+ patients is shown in supplementary Fig.4, where the Pearson correlation coefficients of significant correlations are color-coded in yellow-green scheme. The significance is determined by permutation tests.

**
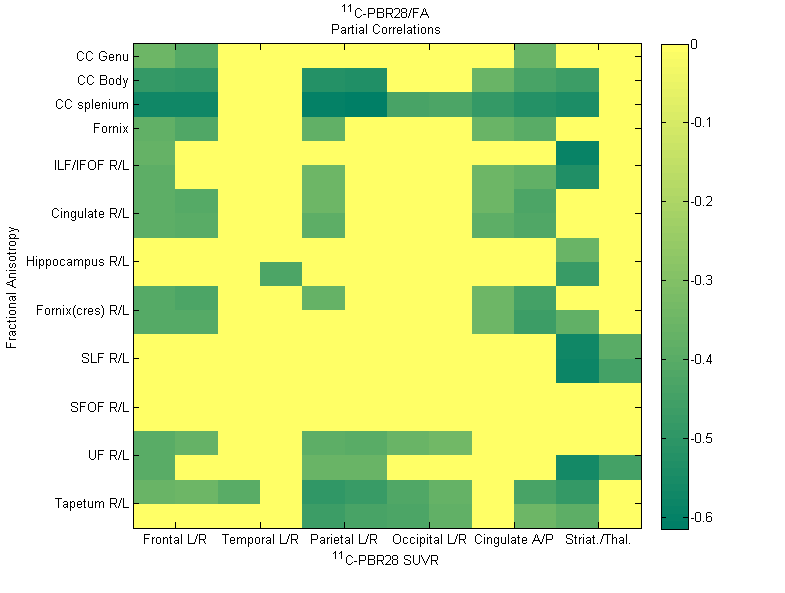
**

**Supplementary Figure 4. Partial correlation matrix between ROIs of ^11^C-PBR28 and FA values in tracts of interest in Aβ-positive patients.**

Correlation coefficients of non-significant correlations are plotted as zeros.

CC: corpus callosum; ILF: inferior longitudinal fasciculus; IFOF: inferior fronto-occipital fasciculus; SLF: superior longitudinal fasciculus; SFOF: superior fronto-occipital fasciculus; UF: uncinate fasciculus; L: left; R: right; A: anterior; P: posterior; Striat: striatum; Thal: Thalamus.

**Supplementary file 7: Model performance of SCCA under different LASSO penalties**

In the current study, a range of λ (from 0.10 to 0.70 incrementing at 2/3) were tested in SCCA analysis. The model fits (Z-statistics) were summarised in Supplementary Fig.5. The canonical correlation between ^11^C-PBR28 uptake and FA values was strong across different λ constraints and the best model performance was obtained with a relatively light regularisation (λ =0.70). The canonical correlation between ^18^F-Flutemetamol uptake and FA values was weak across different λ constraints even in best performing model (λ =0.50). Adding ^18^F-Flutemetamol uptake to ^11^C-PBR28 uptake did not improve the model fit (compared to ^11^C-PBR28 uptake alone), and the best model performance was obtained with a relatively light regularisation (λ =0.70).


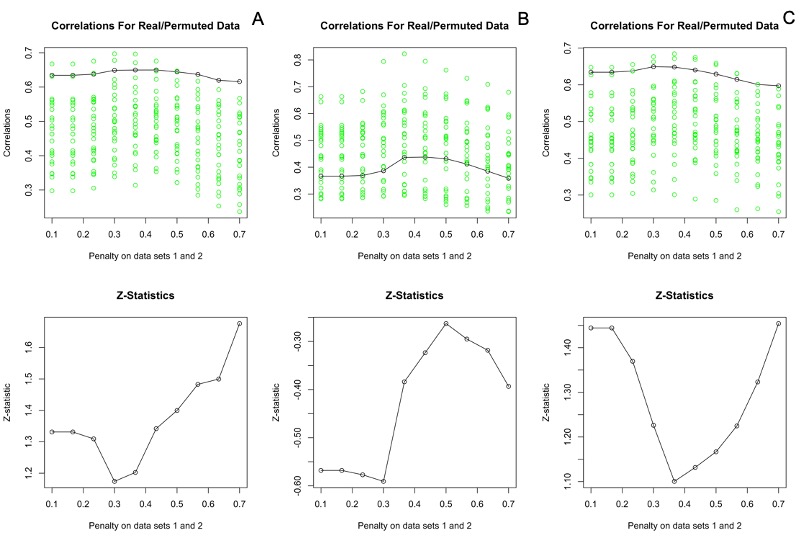


**Supplementary Figure 5. Model Performance of Sparse Canonical Correlation Analyses under Different LASSO Penalties**. A. Model performance in SCCA between ^11^C-PBR28 uptake and FA values under different λ; B. Model performance in SCCA between ^18^F-flutemetamol uptake and FA values; C. Model performance in SCCA between ^18^F-flutemetamol and ^11^C-PBR28 uptake and FA values. The top row shows the position of observed canonical correlation coefficients (points in black) versus the distribution of permuted coefficients (points in green). The bottom row shows the Z-statistics of SCCA under different λ.

**Supplementary file 8: Association between microglial activation and brain network measures in different diagnostic groups**

To test whether microglial activation had stage-dependent relationship with brain structural network integrity at MCI and AD stages, we also performed same linear analysis in the two groups separately, and the results suggested same direction of association, though halved sample size limited the statistical power. *^11^C-PBR28 PC1* had a trend of negative association with both *FA PC1* and *FA PC2* in MCI patients (standardised *β*=-0.64, *p-perm*=0.057; standardised *β*=-0.45, *p-perm*=0.16, corrected for cortical thickness in addition to age and gender). Same trend was found in AD group (standardised *β*=-0.55, *p-perm*=0.08; standardised *β*=-0.12, *p-perm*=0.72, corrected for cortical thickness in addition to age and gender). For local efficiency, *^11^C-PBR28 PC1* had marginal negative influence in both AD and MCI patients (p=0.018 and 0.087, respectively).


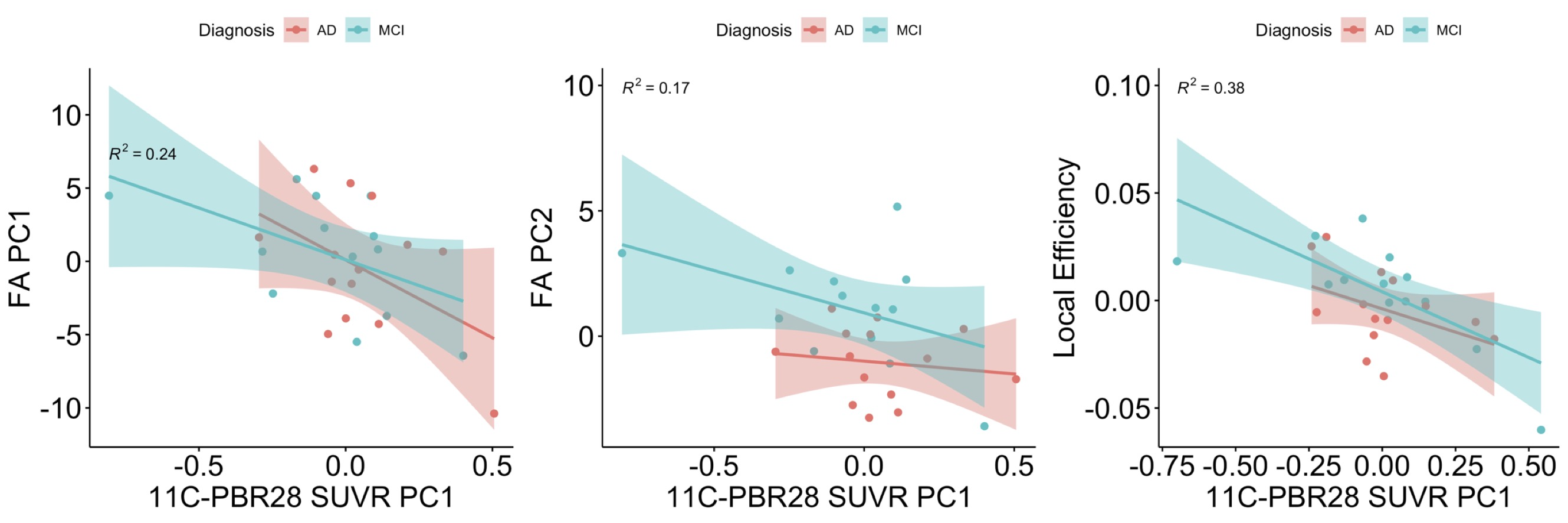


Supplementary Figure 6. Association between 11C-PBR28 uptake and network measures in MCI and AD groups separately.

**Supplementary file 9: Regression analysis without outlier**

As there is a potential outlier in the Aβ+ group (a 75-year-old MCI patient), we have also re-tested the findings without the outlier point, and the results remain largely similar to the original analysis:

In Aβ+ patients, cortical thickness had a positive association with FA PC2 (standardised β=0.48, 95%CI 1.37~0.85, p-perm=0.027, corrected for age and gender), while ^18^F-flutematemol PC1 was not associated with either of the FA PCs, suggesting that association fibre integrity is quantitatively associated with grey matter preservation, but not Aβ plaque load. ^11^C-PBR28 PC1 was negatively associated with FA PC1 (standardised β=-0.52, 95%CI: -0.95~-0.11, p-perm=0.016), but not FA PC2 (standardised β=-0.34, 95%CI: -0.73~0.05, p-perm=0.12, corrected for cortical thickness in addition to age and gender).

^11^C-PBR28 PC1 negatively correlated with Eloc (standardised β=-0.61, 95%CI: -0.94~-0.25, p-perm=0.001, cortical thickness corrected in addition to age and gender), but not Eg nor SW.

FA PC2 had significant positive association with Eloc (standardised β=0.40, 95%CI: 0.03~0.76, p-perm=0.02, thickness, age and gender corrected, Fig.3H), while cortical thickness correlated with SW (standardised β=0.61, 95%CI: 0.24~0.98, p-perm<0.001, PC2, age and gender corrected).

FA PC2, local efficiency and cortical thickness positively correlated with MMSE (standardised β=0.45, 95%CI: 0.06~0.84, p-perm=0.014; standardised β=0.44, 95%CI: 0.05~0.83, p-perm=0.024; and standardised β=0.52, 95%CI: 0.15~0.90, p-perm=0.007, respectively).


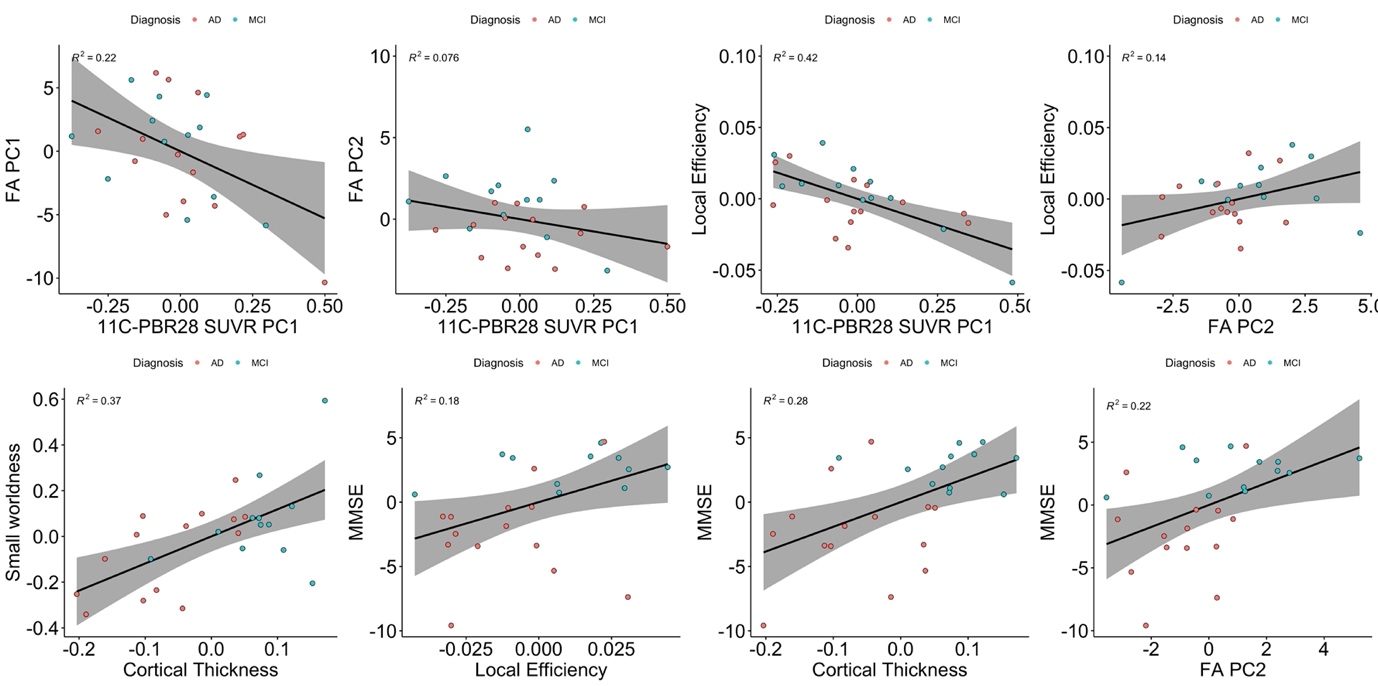


Supplementary Figure 7. Partial regression analysis without the outlier

**Supplementary file 10: Considerations of age**

As there was a significant difference of age between HC and cognitively impaired patients, we have first tested whether age had a strong influence on the network metrics in our healthy control cohort. We did observe a trend of negative association between age and network organisation metrics, but the effect of age was not strong enough to reach significance (Table 2), which suggested that considering the strength of its effect, age should not have confounded our inference seriously. To test the prior hypothesis, we further examined the effect of age in the full cohort where network metrics were defined as dependent variable while age and diagnostic groups were defined as independent variables. We found that indeed, in the group-wise comparisons, age did not have significant weights in the models (Table 3).

Table 2. Correlation coefficients of age in healthy control cohort

| Healthy controls | Age | |
| --- | --- | --- |
| metrics | *standardised β* | *p* |
| Small Worldness | -0.06 | 0.90 |
| Local Efficiency | -0.27 | 0.60 |
| Global Efficiency | -0.27 | 0.61 |
| FA PC1 | -0.31 | 0.55 |
| FA PC2 | -0.63 | 0.18 |
| Cortical Thickness | -0.23 | 0.7 |
| ^11^C-PBR28 PC1 | -0.39 | 0.45 |
| ^18^F-flutemetamol PC1 | 0.15 | 0.78 |

Table 3. Regression coefficients of age in group wise comparison models

| Healthy controls | Age | |
| --- | --- | --- |
| metrics | *standardised β* | *p* |
| Small Worldness | -0.003 | 0.99 |
| Local Efficiency | -0.14 | 0.56 |
| Global Efficiency | -0.09 | 0.72 |
| FA PC1 | -0.31 | 0.32 |
| FA PC2 | -0.32 | 0.17 |
| Cortical Thickness | -0.23 | 0.11 |
| ^11^C-PBR28 PC1 | -0.25 | 0.31 |
| ^18^F-flutemetamol PC1 | 0.24 | 0.15 |

We have performed a 1:1:1 exact match of HC, MCI and AD patients by age (within 12 months), but due to the imbalanced age distribution in each group, we were only able to select 5 participant per group. Here are some of the results: the median ages of participants were 73 (54-76), 74 (56-77) and 72 (55-76) years for AD, MCI and HC groups respectively. And the median MMSE scores were 22 (20-26), 27 (26-29) and 29 (27-30) for these groups. The average ^11^C-PBR28 SUVR in medial temporal lobe were 1.01, 1.03 and 1.11 respectively, and a stepwise functional network organisation disruption could be seen in these participants (Figure below). However, due to the small sample size, no statistical conclusions could be drawn in group-wise comparisons. Negative associations could still be observed between ^11^C-PBR28 uptake and local efficiency in the patients (P=0.038, age, gender, cortical thickness and ^18^F-flutemetamol uptake corrected), and between ^11^C-PBR28 uptake and FA PC2 (p=0.12), though not statistically significant due to limited sample size.


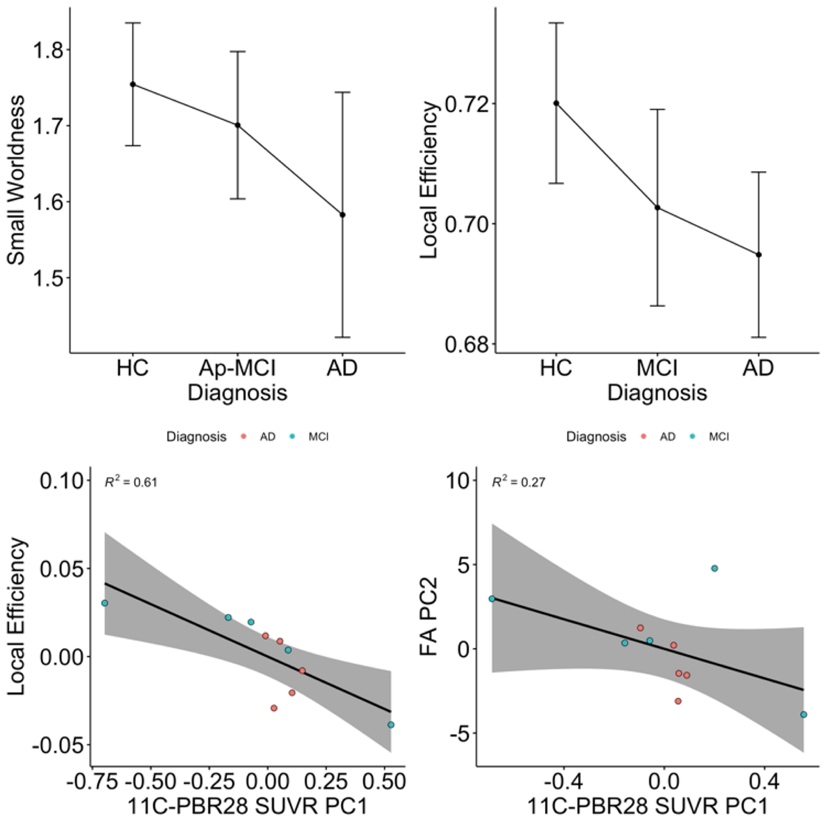


Supplementary Figure 8. Results from 1:1:1 match

1. Rubinov M, Sporns O. Complex network measures of brain connectivity: uses and interpretations. *Neuroimage*. Sep 2010;52(3):1059-69. doi:10.1016/j.neuroimage.2009.10.003

2. Watts DJ, Strogatz SH. Collective dynamics of 'small-world' networks. *Nature*. Jun 4 1998;393(6684):440-2. doi:10.1038/30918

3. Humphries MD, Gurney K. Network 'small-world-ness': a quantitative method for determining canonical network equivalence. *PLoS One*. Apr 30 2008;3(4):e0002051. doi:10.1371/journal.pone.0002051

4. Witten DM, Tibshirani R, Hastie T. A penalized matrix decomposition, with applications to sparse principal components and canonical correlation analysis. *Biostatistics*. Jul 2009;10(3):515-534. doi:10.1093/biostatistics/kxp008
